# Enokitake Mushroom and Its Active Component, Adenosine, Which Restores Testosterone Production in Impaired and Fatigued Mouse Models

**DOI:** 10.3390/nu15092142

**Published:** 2023-04-29

**Authors:** Kazuaki Iguchi, Koji Nagashima, Jun Mochizuki, Hiroyuki Yamamoto, Keiko Unno, Noriyuki Miyoshi

**Affiliations:** 1 Laboratory of Biochemistry, Graduate School of Integrated Pharmaceutical and Nutritional Sciences, University of Shizuoka, Shizuoka 422-8526, Japan; miyoshin@u-shizuoka-ken.ac.jp; 2 TechnoSuruga Laboratory Co., Ltd., Shizuoka 424-0065, Japan; koji.nagashima@tecsrg.co.jp (K.N.); jm_1001@tecsrg.co.jp (J.M.); 3 Department of Microbiology and Molecular Cell Biology, Nihon Pharmaceutical University, Saitama 362-0806, Japan; yamamoto@nichiyaku.ac.jp; 4 Tea Science Center, Graduate School of Integrated Pharmaceutical and Nutritional Sciences, University of Shizuoka, Shizuoka 422-8526, Japan; unno@u-shizuoka-ken.ac.jp

**Keywords:** testosterone, adenosine, enokitake, mushroom, vegetable, *Cordyceps*, cordycepin, testis, cisplatin, stress, fatigue

## Abstract

Several studies have reported the effects of the consumption of various mushroom species on the testes in animal experimental models. Mushrooms, including enokitake mushrooms (*Flammulina velutipes*), and vegetables contain adenosine may affect testosterone production. Here, we aimed to elucidate the effects of enokitake and its active component, adenosine, on testosterone production in primary cultures of testicular cells in vivo using mice models and in vitro. The administration of enokitake ethanolic extract increased testosterone production in the cisplatin-impaired mouse model. The direct effect of mushroom extracts on testicular cells was examined and liquid chromatography–mass spectrometry analysis confirmed that the mushroom- and vegetable-induced increase in testosterone production mainly involved adenosine. Additionally, the administration of enokitake extract or adenosine to wet floor fatigue model mice promoted testicular testosterone production and enhanced Leydig cell function through insulin-like peptide three level upregulation. Structurally related compounds, including cordycepin, showed lower bioactivity than adenosine. This study showed that the ingestion of adenosine-containing mushrooms and vegetables may effectively increase testicular testosterone production. We conclude that mushrooms with a relatively high adenosine content, such as enokitake, may be useful against aging and fatigue.

## 1. Introduction

Many plants, including mushrooms, are used in ancient, traditional folk medicine as tonic herbs to help restore, tone, and invigorate systems in the body or promote general health and wellbeing. Several herbs are known to relieve fatigue and increase energy. The effects of such tonic herbs are presumably related to androgens and are regarded as energy enhancers. Fatigue is one of the symptoms of male menopause as androgen levels decline with age. Various traditional folk medicines have been used to enhance metabolism to reduce fatigue [1,2,3]. In particular, mushrooms and vegetables contain adenosine which may affect testosterone production. However, a systematic review by Smith et al. [4] showed that most natural products from plants, herbs, and seeds tested against androgen deficiency in human trials failed to increase testosterone levels. Conversely, fenugreek seeds [5] and ashwagandha [6] increase testosterone levels but the active ingredients in them have not been identified. In experimental studies using animals and extracts of various plants such as *Ruta chalepensis* [7], *Lycium barbarum* [8], *Hibiscus macranthus* [9], *Massularia acuminata* [10], *Piper guineense* [11], and *Tribulus terrestris* [12] increased testosterone levels [13,14]; however, the bioactive ingredients responsible for this testosterone-increasing effect remain unknown.

As for mushrooms, testosterone-increasing effects have been reported for the extracts of *Cordyceps sinensis* [15,16], *Paecilomyces tenuipes* [17], *Pleurotus tuber-regium* [18,19,20], and *Morchella esculenta* [21]. Specifically, in *Cordyceps sinensis*, cordycepin is known to be the active ingredient responsible for increasing testosterone levels [22]. These mushrooms are mainly used for medicinal purposes and are not commonly found. Edible mushrooms have been shown to contain different bioactive components with medicinal properties [23]. Namely, enokitake (*Flammulina velutipes*), also known as a golden needle or winter mushroom, is one of the most popular edible mushrooms with various bioactivities [24]. In this study, we aimed to elucidate the effects of enokitake and its active component, adenosine, on testosterone production using both impaired and fatigue mouse models as well as in vitro primary cultured mouse testicular cells.

## 2. Materials and Methods

### 2.1. Chemicals

Adenosine, cordycepin, and related nucleosides were purchased from Tokyo Kasei Kogyo (Tokyo, Japan). Cisplatin (Cp) was purchased from Wako Pure Chemical (Osaka, Japan). Collagenase type 2 was purchased from Worthington Biochemicals (Freehold, NJ, USA). 5′-Methylthioadenosine, *S*-adenosylmethionine, human chorionic gonadotropin (hCG), fetal bovine serum (FBS), Dulbecco’s Modified Eagle (DME)/F-12 medium, testosterone-related steroids, and the reagents for cell culture were obtained from Sigma-Aldrich (St. Louis, MO, USA). The other reagents used were obtained from Wako Pure Chemical or Sigma–Aldrich and were of special or biochemical grade.

### 2.2. Preparation of Mushroom and Other Extracts

Mushrooms (enokitake [*F. velutipes*], shiitake [*Lentinus edodes*]), vegetables (parsley [*Petroselinum crispum*], Chinese cabbage [*Brassica rapa* var. *glabra*], and spinach [*Spinacia oleracea*]) were obtained from local markets in Japan. The enokitake mushrooms used were of the cultivated version: brownish, wild type, and commonly known as “*Ezoyukinoshita*” (Hokkaido, Japan). Reishi mushrooms (*Ganoderma lucidum*) were purchased from Ishihara Sangyo (Aichi, Japan). *Cordyceps militaris* (product name, Hokuchusou; cultivated type, China) and *C. sinensis* (product name, Kinchuso; Tibet, China) were obtained in Japan. Each sample was air-dried at 60 °C, except for reishi and *Cordyceps* mushrooms, which were obtained as dried samples. The samples were powdered in a food processor and extracted with ethanol. Briefly, the powdered samples were added to 30% ethanol (0.5 mg dry powder/mL), mixed overnight at room temperature (15–25 °C), and centrifuged at 7000× *g* for 15 min. The supernatants were passed through 70-μm nylon filters (Corning Inc., Corning, NY, USA) and frozen until use. Enokitake was also extracted with 50% and 80% ethanol following the procedure described above.

### 2.3. Administration of Enokitake Extract and Adenosine to Animal Models

Two animal models were used in the experiments described herein:

(1) Cp-impaired model. Mice (ddY, male, 7-week-old; Japan SLC Inc., Shizuoka, Japan) were assigned to three groups (four mice/group) as follows. In experiment 1, mice in the Cp and Cp + Ee groups were intraperitoneally administered Cp (0.2 mg/saline/mouse) on day 5. Mice in the Cp + Ee group were provided drinking water containing 6% of 30% ethanol extract of enokitake. Mice in the control group were provided water containing 1.8% ethanol without Cp. After 14 d, mice were euthanized at 09:00–11:00 h and the testes were dissected for culture and quantitative polymerase chain reaction (qPCR). In experiment 2, the Cp and Cp + Ado group mice were intraperitoneally administered Cp (0.2 mg/saline/mouse). Mice in the Cp + Ado group were provided drinking water containing 0.01% adenosine. Mice in the control group were provided water. After 12 d, the mice were euthanized at 09:00–11:00 h and the testes were dissected for culture and qPCR.
(2) The wet floor fatigue model: Mice (ddY, male, 7-week-old; Japan SLC Inc.) were assigned to two groups (four mice/group) and housed in individual cages on wood chips. The test group was allowed to drink 1:20 diluted 30% ethanol extract of enokitake or 0.1% adenosine solution for 10 d. The mice were transferred to wet floor cages on day 6. A soft plastic plate was wrapped in six paper towels moistened with water and placed at the bottom of the cages to create a wet floor, and the mice were housed in these cages for 24 h. The amount of water was adjusted to ensure that the extremities of the mice were submerged to a depth of <5 mm, and chow was provided *ad libitum*. Mice were returned to the same individual cages. After 3 d, the mice were euthanized at 09:00–11:00 h, and the testes were dissected for culture and qPCR.

In all experimental groups, mice were fed the same diet (MF, Oriental Yeast Co., Tokyo, Japan) under conventional conditions in a temperature and humidity-controlled room with a 12-/12-h light–dark cycle (lights on at 08:00 h). Experiments were performed in accordance with the Guidelines for the Regulation of Animal Experimentation Committee of the University of Shizuoka (Approval Nos. 156154 and 186330).

### 2.4. Effect of the Extracts and Compounds on the Primary Culture of Mouse Testicular Cells

The in vitro effects of the extracts and adenosine-related compounds (cordycepin, 5′-methylthioadenosine, *S*-adenosylmethionine guanosine, inosine, adenine, and caffeine) on testicular testosterone production were examined in primary cultures of mouse testicular cells prepared using the method of Yang et al. [25]. Briefly, the testes of ddY mice (male; age, 7–11 weeks; Japan SLC Inc.) were excised, decapsulated, and incubated in medium 199 (Sigma-Aldrich) containing 0.4% collagenase and 0.1% bovine serum albumin (BSA) at 37 °C for 15 min. The testes were cooled, passed through a 70-μm nylon mesh (Corning Inc.), and centrifuged at 450× *g* for 15 min at 4 °C. The cells were washed with DME/F-12 medium and seeded in DME/F-12 containing 10% FBS at a density of 3 × 10^5^ cells/well in a 24-well plate (Primaria, Corning Inc.). After incubation for 18–24 h, the cells were washed twice with DME/F-12 and incubated with diluted extracts or compounds in DME/F-12 containing 0.1% BSA for another 2–3 h and the medium was collected and subjected to enzyme immunoassays (EIAs) for the measurement of testosterone. The testes obtained from the experiments described in Section 2.3 were also used without stimulants. The validation of primary culture preparation was performed using hCG as a positive control.

### 2.5. EIAs

EIAs for the measurement of testosterone were established using anti-testosterone serum and biotinylated testosterone. Testosterone antiserum was prepared via immunization with a testosterone-BSA conjugate using the *N*-succinimidyl ester method [26]. Briefly, testosterone-3-(O-carboxymethyl) oxime (7.2 mg) was coupled with BSA (20 mg) using hydroxysuccinimide (2.3 mg) and dicyclocarbodimide (4.1 mg). The lyophilized conjugate was dissolved in saline and emulsified with equal volumes of complete Freund’s adjuvant. The emulsion was then intradermally injected into female rabbits (Japanese white rabbits, Japan SLC Inc.) 3 times at intervals of 2–3 weeks. The blood was collected from the ear vein to obtain serum and then the titer was measured using the testosterone-coated microplate enzyme-linked immunosorbent assay. Biotinylated testosterone was prepared as a tracer following the method described by Dressendörfer et al. with slight modifications [27]. Briefly, testosterone 3-(O-carboxymethyl) oxime (3.6 mg) in DMF was reacted with *N*-hydroxysuccinimide (1.1 mg) and dicyclohexylcarbodimide (2.0 mg) for 24 h. After removing undissolved material via filtration, the active ester was reacted with 6-[6-(biotinyl-amino) hexanoylamino] hexanoylhydrazine (4.85 mg) for 48 h. The reaction mixture was separated via reverse-phase high-performance liquid chromatography and the fraction containing biotinylated testosterone was detected based on the binding ability to anti-testosterone antiserum.

The standard diluent used as an EIA buffer included 10 mM phosphate-buffered saline containing 25 mM ethylenediaminetetraacetate and 0.04% Tween 20 (pH 7.4). Subsequently, 2 µg of goat anti-rabbit IgG purified using the HiTrap Protein G HP column (GE Healthcare, Uppsala, Sweden) was adsorbed onto a 96-well microplate (Nunc, Thermo Fisher Scientific, Waltham, MA, USA). After washing with water containing 1% BSA, 0.01 M phosphate-buffered saline was added to the plates which were then set aside for 15 min. After washing with 0.02% Tween 20, the samples were incubated with serially diluted standard testosterone or appropriately diluted samples, anti-testosterone serum (1:540,000 diluted), and biotinylated testosterone (2 pg) for 1 h. Peroxidase-conjugated streptavidin (1:5000 diluted; Jackson ImmunoResearch Laboratories, West Grove, PA, USA) was added to the washed microplates. After incubation for 1 h, the microplates were washed twice with 0.04% Tween 20 and *o*-phenylenediamine solution was added. The color was allowed to develop for 10 min and the absorbance at 490 nm was measured using a MultiSkan FC spectrometer (Thermo Fisher Scientific). The cross-reactivity of testosterone structure-related androgen steroids in the EIA with standard testosterone was <0.1%, except for 4-androstene-3,17-dione, which showed a cross-reactivity of 5.05%.

### 2.6. Determination and Quantification of Adenosine in Enokitake via LC–MS

The enokitake extract was desalted using a D-salt dextran desalting column (Pierce Dextran Desalting Columns; Thermo Fisher Scientific); bioassays were performed to monitor testosterone release in testicular primary cell preparations. Briefly, 30% enokitake ethanol extract was evaporated, dissolved in water, and applied to the desalting column. The void bioactive peak fraction was collected and subjected to purification using an SPE column (EVOLUTE ABN, 60 mg absorbent mass; Biotage, Uppsala, Sweden), prewashed with methanol and then with 0.1% formic acid, and eluted using a stepwise gradient of water and methanol. Each fraction was mixed with ×4 volumes of methanol and centrifuged to remove glycan. Subsequently, an aliquot of the supernatant was injected into a liquid chromatography–mass spectrometry (LC–MS) system consisting of an AQUITY ultra-performance liquid chromatography (UPLC) system (Waters, Milford, MA, USA) coupled with a micrOTOFQII mass spectrometer (Bruker Daltonics, Bremen, Germany) [28,29]. Chromatographic separation was performed using a BEH C18 column (1.7 μm, 50 mm × 2.1 mm i.d., Waters) at 40 °C. Samples were eluted from the column using solvent A (0.1% formic acid in ultrapure water) and solvent B (0.1% formic acid in acetonitrile) via a linear gradient of 1% solvent B from 0 to 3 min to 80% solvent B at 20–24 min. The flow rate of the mobile phase was 0.4 mL/min. The time-of-flight mass spectrometry (TOF–MS) was operated in the positive and negative ion modes using an electrospray ionization source. MS peak data from UPLC–TOF–MS analyses were subjected to Compass Data Analysis (Bruker Daltonics). The adenosine level in samples was estimated using external standard methods.

### 2.7. qPCR

Total cellular RNA was isolated from mouse testes using RNAiso Plus (Takara, Shiga, Japan). cDNA was synthesized from the total RNA using ReverTra Ace (Toyobo, Tokyo, Japan) with random primers and dNTPs (Nippon gene, Tokyo, Japan) based on the protocol by the manufacturer. Real-time qPCR was performed in the Light Cycler Nano system (Roche Diagnostics, Mannheim, Germany) using SYBR green RT–PCR reagent (Toyobo) following the protocol by the manufacturer. Primers for mouse insulin-like peptide 3 (*Insl3*) and β-actin mRNA were designed using Primer3plus online software and prepared via custom synthesis (Sigma Genosys, Hokkaido, Japan). Primer sequences used were as follows: *Insl3*: 5′-CTACTGATGCTCCTGGCTCTGG-3′ (forward) and 5′-GAGATGTCTCTGCTCTAGCCAC-3′ (reverse); β-actin: 5′-CACCTTCTACAATGAGCTGCGTG-3′ (forward) and 5′-ATGGCTGGGGTGTTGAAGGTCT-3′ (reverse). The *Insl3* mRNA level was normalized to that of *β*-actin mRNA in each sample.

### 2.8. Statistical Analysis

Data are expressed as means ± standard error of the means. The Student’s *t*-test or the Tukey–Kramer’s multivariate comparison test was used to determine significant differences among treatment means. The differences were significant at *p* < 0.05.

## 3. Results

### 3.1. Enokitake Extract and Adenosine Treatment

#### 3.1.1. Effect of Enokitake Extract or Adenosine on Testicular Testosterone Production

**Figure 1:**
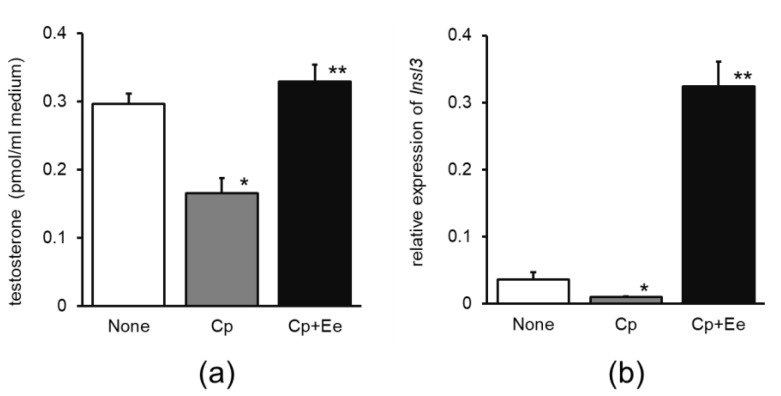
Effects of enokitake extract administration on (**a**) testosterone release and (**b**) relative expression of *Insl3* mRNA in primary cultures of mouse testicular cells. None, water without cisplatin treatment; Cp, cisplatin treatment alone; Cp + Ee, enokitake extract administration with cisplatin treatment. Mean ± standard error, n = 4, * *p* < 0.05, compared to None, ** *p* < 0.05, compared to Cp. *Insl3*, insulin-like peptide three.

Testosterone production in primary cultures of testicular cells was significantly reduced by Cp treatment compared with that in the control group and 30% ethanol extract of enokitake strongly reversed this decrease in testosterone production (Figure 1a). *Insl3* mRNA expression was 0.27-fold lower in the Cp group and 33-fold higher in the Cp + Ee group than in the control group (Figure 1b).

**Figure 2:**
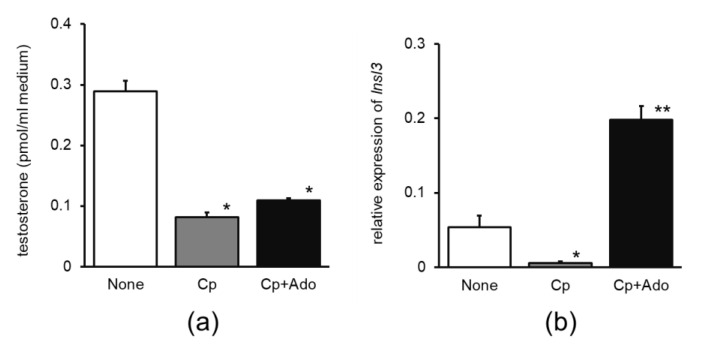
Effects of adenosine administration on (**a**) testosterone release and (**b**) relative expression of *Insl3* mRNA in the primary cultures of mouse testicular cells. None, water without cisplatin treatment; Cp, cisplatin treatment alone; Cp + Ado, adenosine administration with cisplatin treatment. Mean ± standard error, n = 4, * *p* < 0.05, compared to None, ** *p* < 0.05, compared to Cp. *Insl3*, insulin-like peptide three.

The increase in testosterone production in the Cp + Ado group was slightly higher than that in the Cp group (Figure 2a); *Insl3* mRNA expression increased considerably in the Cp + Ado group compared to that in the Cp group (Figure 2b).

#### 3.1.2. Direct Effect of Enokitake Extract on Testosterone Production in Primary Cultures of Testicular Cells

**Figure 3:**
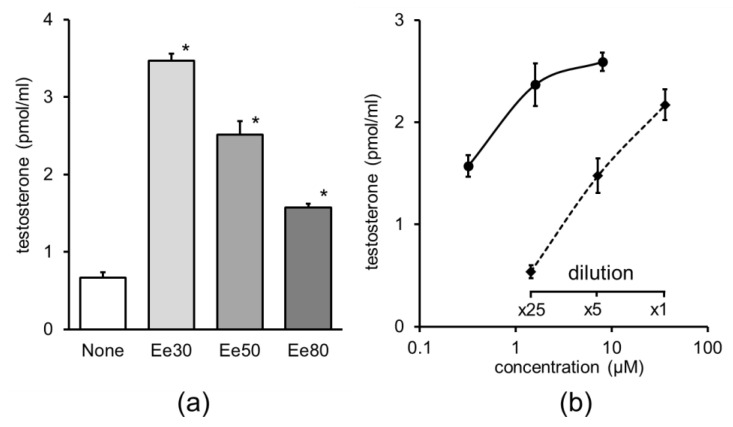
Effect of enokitake extracts on testosterone production in primary cultures of testicular cells. The extract used corresponds to 5 mg of dry powder of enokitake; mean ± standard error, n = 4, *, compared to none, *p* < 0.05. (**a**) Enokitake ethanolic extract diluted (1:100) with culture medium; Ee30, 30% ethanol extract; Ee50, 50% ethanol extract; Ee80, 80% ethanol extract. (**b**) Dose–response curves of adenosine and dilution curve of 30% ethanol extract of enokitake. Solid curve, adenosine; dashed curve, enokitake extract. Mean ± standard error, n = 4.

The addition of 30%, 50%, or 80% enokitake ethanolic extract considerably increased testosterone production in primary cultures of testicular cells and the 30% ethanol extract was the most effective (Figure 3a). Serial dilution of the 30% enokitake extract was found to dose-dependently increase testosterone production and its dilution curve was almost parallel to that of adenosine (Figure 3b).

### 3.2. LC–MS Analysis of Enokitake Extract

The enokitake ethanolic extract was applied to a D-salt column and the effect of the fractions on testosterone production in testicular primary culture cells was evaluated. The bioactive void fraction was then separated using an SPE column. The most bioactive fraction eluted with 20% ethanol was collected and analyzed using LC–MS/MS. The prominent peak component corresponding to 268.0997 *m/z* was identified as adenosine via database analysis. The adenosine-mediated increase in testosterone production by primary testicular cells was confirmed to be dose-dependent (Figure 3b).

### 3.3. Effect of Enokitake Extract or Adenosine on Testicular Testosterone Production in the Wet Floor Stress Model

**Figure 4:**
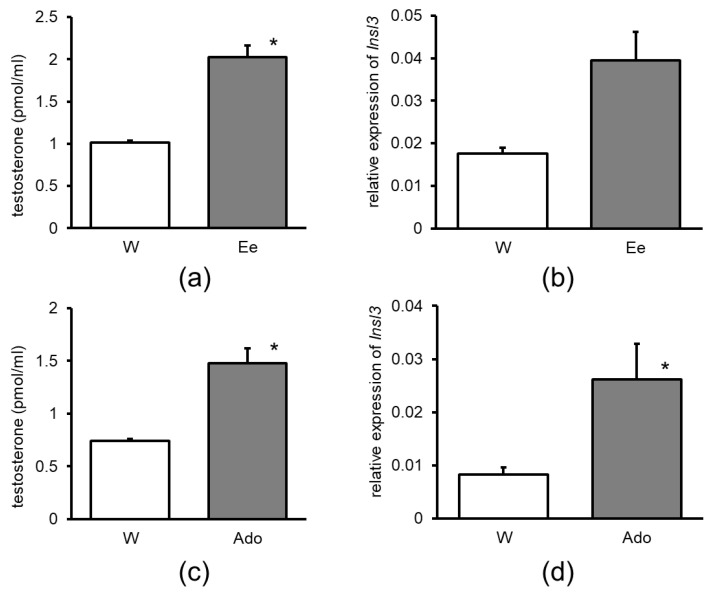
Effect of the administration of 30% ethanol extract of enokitake on (**a**) testicular testosterone production and (**b**) testicular *Insl3* mRNA expression. Effect of the administration of Ado on (**c**) testicular testosterone production and (**d**) testicular *Insl3* mRNA level in the wet floor model mice. W, water; Ee, enokitake extract; Ado, adenosine; *Insl3*, insulin-like peptide three. Asterisks indicate a significant difference compared to water, *p* < 0.05.

The group of mice that was administered enokitake ethanolic extract or adenosine showed a significant increase in testosterone production compared to the control group (Figure 4a,c). Furthermore, *Insl3* mRNA expression significantly increased upon treatment with both enokitake ethanolic extract and adenosine (Figure 4b,d).

### 3.4. Correlation of Adenosine Concentration and the Effect of the Extracts of Mushrooms and Vegetables on Testosterone Production

**Figure 5:**
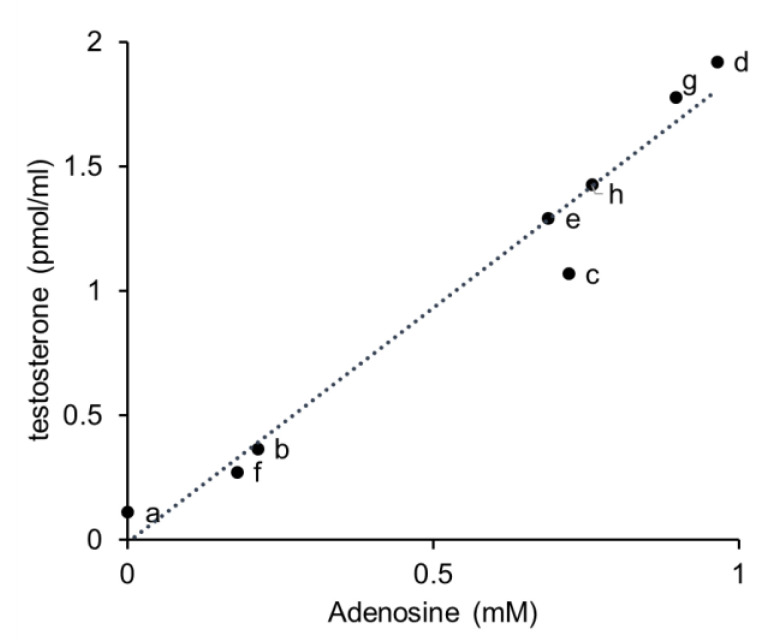
Correlation between adenosine concentration and the effect of mushroom and vegetable extracts on testosterone production. The dotted line represents linear regulation in curve plots. a, parsley; b, Chinese cabbage; c, spinach; d, enokitake; e, shiitake; f, reishi; g, *Cordyceps sinensis*; h, *Cordyceps militaris*.

The concentration of adenosine in mushrooms and vegetables was determined using LC–MS and testosterone production was measured in primary cultures of testicular cells. A strong relationship between adenosine concentration and testosterone production was observed; indeed, the correlation coefficient of the regulation curve was as high as 0.9825 (Figure 5). Relatively high concentrations of adenosine were detected in mushrooms. Cordycepin was also detected in the extract of *Cordyceps* mushrooms via LC–MS (data not shown).

### 3.5. Effect of Adenosine Structure-Related Compounds on Testosterone Production

**Figure 6:**
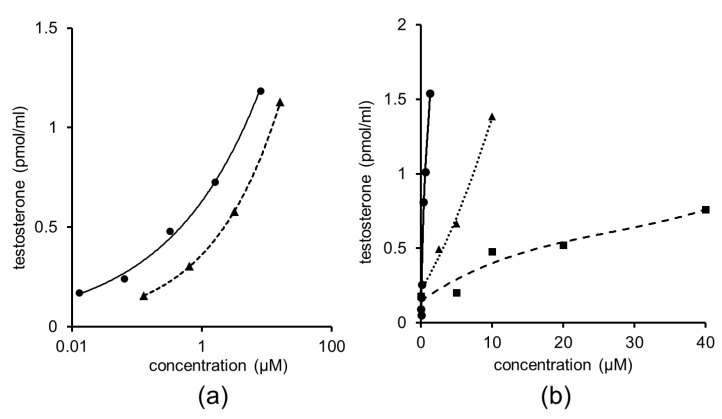
Effect of treatment with serially diluted adenosine-related compounds on testicular testosterone production in primary cultures of testicular cells. (**a**) Solid curve, adenosine; dashed curve, cordycepin. (**b**) Solid curve adenosine; dotted curve, 5′-methylthioadenosine; dashed curve, *S*-adenosylmethionine.

Cordycepin, 5′-methylthioadenosine, and *S*-adenosylmethionine affect testosterone production considerably. The dose–response curve of cordycepin was almost parallel to that of adenosine and the relative activity of adenosine was calculated as 0.18 (Figure 6a). Furthermore, 5′-Methylthioadenosine and *S*-adenosylmethionine induced low testosterone production and their curves were not parallel to the curve of adenosine (Figure 6b). No significant increase in testosterone production was observed upon treatment with adenosine structure-related compounds, including guanosine, inosine, and adenine at 100 µM.

## 4. Discussion

We investigated the effects of enokitake mushrooms and their active component, adenosine, on testosterone production in mice. The administration of enokitake ethanolic extract significantly restored testosterone production in cisplatin-treated mouse testes. We established an impaired testicular mouse model using cisplatin which has been shown to generate reactive oxygen species (ROS) that cause testicular damage [30,31,32]. Enokitake contains various polyphenols (e.g., chlorogenic acid, rutin, quercetin, gallic acid, and ferulic acid) which may scavenge these ROS [33,34,35]. The ameliorative effect of polyphenols in natural products on the testes has been reported [36,37,38]. Furthermore, the increase in testosterone production attained upon treatment with 30% enokitake ethanolic extract was higher than that resulting from treatment with the 80% extract, suggesting that highly water-soluble polyphenols (e.g., their glycosylated derivatives) in enokitake contributed to the observed enokitake-mediated increase in testosterone production.

Adenosine, which was found in the 30% ethanol enokitake extract, showed low testosterone-release activity in the Cp-impaired model under the conditions tested. Conversely, in vitro experiments with adenosine showed a significant increase in testosterone production, strongly suggesting that the effect of the enokitake ethanolic extract might be attributed to adenosine. Consistently, a nucleoside-rich extract of *Cordyceps* prevents Cp-induced testicular damage [39].

The increase observed in *Insl3* mRNA expression in impaired testes contributed to the recovery of testicular function. Specifically, *Insl3* is expressed in Leydig cells which produce testosterone and its expression is strongly associated with Leydig cell function [40,41]. Considering *Insl3* mRNA expression, active constituents such as adenosine in enokitake extracts can apparently act on Leydig cells.

In this study, enokitake extract or adenosine was mixed with drinking water and administered orally. Certain nucleoside transporters involved in the absorption of adenosine are present in the small intestine [42,43,44], indicating that adenosine may directly act on the testes. Here, chromatographic separation of the 30% extract of enokitake revealed that the most probable active component was adenosine, which exhibits steroidogenic activity [45,46]. The dose–response curves of the adenosine and enokitake extract-mediated increase in testosterone production were almost parallel, indicating that the active component of the enokitake ethanolic extract is very likely adenosine. Cordycepin, also known as 3′-deoxyadenosine which is a specific constituent of *Cordyceps*, has been investigated for its bioactivity [47]. Indeed, *Cordyceps* spp. induce testosterone production [17,18,48,49]. However, our study provided evidence that the cordycepin-induced production of testosterone may be lower than that of adenosine, indicating that the effect of *Cordyceps* on testosterone production might be attributed to the synergistic effects of cordycepin and adenosine, which is also present in *Cordyceps* [50].

The 30% mushroom and vegetable extracts tested herein were found to contain adenosine and exhibit testosterone production-inducing activity. Furthermore, their relationship exhibited good linearity, indicating that the extent of testosterone production-inducing activity may depend on the specific adenosine content which is commonly present in the extracts of natural products such as mushrooms and vegetables.

We found adenosine structure-related nucleosides, 5′-methylthioadenosine and adenosylmethionine, to have significant but low testosterone production-inducing activity compared with that of adenosine, indicating that nucleosides contribute to the activity of enokitake extract. By contrast, guanosine, inosine, and adenine did not show testosterone production-inducing activity. Meanwhile, adenosine receptors are well known to exist in testes [51,52,53] and presumably affect the testosterone production-inducing activity of adenosine. As observed in this study, mushrooms have a higher adenosine content than other vegetables and their relatively high testosterone production-inducing activity may have anti-aging effects.

Here, the administration of enokitake ethanolic extract increased testosterone production in the fatigue model and adenosine supplementation showed the same effect. Consistently, *Insl3* upregulation in the testes further supported this activity. Our results showed that the enokitake ethanolic extract and its active component, adenosine, effectively promoted recovery from fatigue. The wet floor fatigue model designed herein was slightly modified from the rat fatigue model first created by Tanaka et al. [54]. In the first 24 h, mice tried to avoid water and had little rest or sleep. Upon return to regular cages, the testicular recovery was evaluated after three days. As fatigue is promoted by stress, the supplementation of enokitake or adenosine may be effective in ameliorating stress-related symptoms. As the mouse fatigue model established in this study may simulate the testes’ functional decline caused by social stress in humans, we hypothesize that enokitake and other mushrooms might be used to relieve fatigue and stress.

*Cordyceps* species are special fungi that parasitize insect larvae [55]. Cordycepin (3-deoxyadnosine), a natural analog of adenosine and a specific component found in *Cordyceps*, enhances the testes’ function [22,56]. As mushrooms often contain specific bioactive components, pharmacological effects are expected [57]. In this study, we demonstrated the effects of adenosine and structurally related substances on testosterone production in primary testicular cultured cells. 

Tonic plants and herbs have been investigated for their effects on testosterone production in animal studies [13,14]. Although several herbs have been shown to increase testosterone production, there is no clear evidence for the use of any of the substances identified as their active ingredients, with the exception of cordycepin [22]. Mushrooms cultivated for food, such as enokitake, were also found to increase testosterone production in this study. Concomitantly, adenosine, a component of enokitake mushroom extract, was also found to increase testosterone production. As vegetables contain adenosine, it is important to know their effects on testosterone production.

Testosterone production is well known to decline with aging [1,2]. As this decrease is considered to be related to changes in the testis, we adopted a Cp-induced testicular injury model in this study [58]. Furthermore, one major factor of fatigue is stress, which is thought to cause testis dysfunction [3]; thus, the wet floor model was considered suitable for this study, as water is a strong stressor and can cause fatigue in mice [54]. The testosterone production-increasing effect of enokitake mushroom extract is presumably effective in promoting recovery from stress and stress-induced fatigue. Our observation that enokitake consumption in these animal models of fatigue and aging increased testosterone production indicates that enokitake may be effective for fatigue recovery and as an anti-aging supplement in humans. Among fungi, mushrooms, which have been widely consumed as food, are advantageous in terms of safety when searching for functional food materials. Several herbal and plant-derived tonic traditional medicines, including those that have been shown to increase testosterone production in animals, have not been as effective in humans [4]. Therefore, the application of enokitake or its ethanolic extract in humans should be thoroughly investigated in terms of effectivity and safety. Additionally, the effects of long-term continuous ingestion of enokitake mushrooms in terms of any positive effects it may have, are unknown. Although enokitake mushrooms have been cultivated as food for a long time and have been consumed frequently, to our knowledge, there are no reports of beneficial effects on gonadal function, nor have there been any reports of any notable adverse effects. Adenosine, which is found in enokitake mushrooms, is also present in many foods that are consumed on a daily basis. Furthermore, the potential side effects of enokitake mushrooms or adenosine are difficult to elucidate because adenosine is present in most body fluids and its receptors are widely distributed [59,60]. Lastly, adenosine has been reported to have pro-inflammatory effects on testes [61], but the underlying mechanism of action involved awaits elucidation.

Leydig cells have adenosine receptors and the adenosine analog cordycepin increases testosterone production in Leydig cell cultures [47]. This finding supports the possibility that the effect of adenosine in increasing testosterone production, as revealed in this study, directly acts on Leydig cells. Nonetheless, the presence of adenosine receptors in Leydig cells has not been confirmed by other reports and is rather negative [62]. One of the adenosine receptors, the A1 receptor, is present in germ cells and the A3 receptor in Sertoli cells [53,63]. Several paracrine interactions have been suggested between Leydig and Sertoli cells [64,65]. In this study, using testis cells including Leydig cells, we found that adenosine increased testosterone production. However, this result does not exclude the possibility that adenosine might not act directly on Leydig cells. For example, Rommerts also found evidence that primary cultures of Leydig cells modified the in vitro responsiveness of the cells to adenosine after preparation [46]. Leydig cells have P2-purinergic receptors and extracellular ATP increases testosterone secretion [66,67]. Exoenzymes involved in ATP degradation exist outside the cell and are responsible for the rapid degradation of ATP [68]. However, it is not known how the adenosine produced by the breakdown of ATP affects these enzymes. Extracellular ATP was absent in the primary culture conditions used in this study. This indicates that increased testosterone production is less associated with ATP. Our study clearly showed that adenosine acted directly or indirectly on testicular Leydig cells. To support this finding, the elucidation of the mechanism of action of adenosine on Leydig cells is essential for applications such as to functional food.

## 5. Conclusions

We clarified the effect of the enokitake ethanolic extract on testosterone production both in vivo and in vitro and confirmed that the main active ingredient present in the extract is adenosine which is a ubiquitous biological component whose activity has long been studied. However, regarding enokitake and other edible mushrooms, the effects of adenosine on the testes have not been previously investigated. The enokitake ethanolic extract effectively increased testosterone production and notably contributed to the effective restoration of testicular function impaired by fatigue and aging, as demonstrated in our mouse experimental models. Altogether, our data indicate that mushrooms containing relatively high adenosine levels may be useful against fatigue and aging in humans, which warrants further research.

## Data Availability

No new data were created or analyzed in this study. Data sharing is not applicable to this article.
